# Development and Evaluation of a Cost-Effective, Carbon-Based, Extended-Release Febuxostat Tablet

**DOI:** 10.3390/molecules29194629

**Published:** 2024-09-29

**Authors:** Israa Hamid Al-Ani, Mohammad Hailat, Dina J. Mohammed, Sina Mahmoud Matalqah, Alaa Azeez Abu Dayah, Bashar J. M. Majeed, Riad Awad, Lorena Filip, Wael Abu Dayyih

**Affiliations:** 1 Faculty of Pharmacy, PDRC, Al-Ahliyya Amman University, Amman 19328, Jordan; phdinajm@gmail.com (D.J.M.); smatatqah@ammanu.edu.jo (S.M.M.); alaa.a.abudayah@gmail.com (A.A.A.D.); esolayman@ammanu.edu.jo (B.J.M.M.); 2 Faculty of Pharmacy, Al-Zaytoonah University of Jordan, Amman 11733, Jordan; m.hailat@zuj.edu.jo; 3 School of Pharmaceutical Sciences, Universiti Sains Malaysia, Penang 11880, Malaysia; 4 Faculty of Pharmacy and Medical Sciences, University of Petra, Amman 11196, Jordan; rawad@uop.edu.jo; 5 Faculty of Pharmacy, Iuliu Hatieganu University of Medicine and Pharmacy, 400012 Cluj-Napoca, Romania; lfilip@umfcluj.ro; 6 Faculty of Pharmacy, Mutah University, Al-Karak 61710, Jordan

**Keywords:** FEB, extended release, bioavailability, charcoal, tablet

## Abstract

This study outlines the development of a cost-effective, extended-release febuxostat (FEB) tablet using activated charcoal as an adsorbent to enhance drug release. FEB, a BCS Class II drug, presents formulation challenges due to low solubility and high lipophilicity. We evaluated eight formulations with varying FEB-to-charcoal ratios using FTIR and DSC for physical interactions and followed USP standards for overall assessment. The optimal 1:0.25 FEB-to-charcoal ratio demonstrated a consistent 12 h zero-order release pattern. In vivo studies indicated a significantly extended plasma profile compared to immediate-release tablets. The optimal tablets demonstrated acceptable hardness and disintegration times. This innovative approach enhances patient compliance, improves bioavailability, and reduces production costs, offering a promising solution for controlled FEB delivery.

## 1. Introduction

Febuxostat (FEB) is a non-purine selective inhibitor of xanthine oxidase, widely used for treating hyperuricemia in gout patients. Despite its efficacy, febuxostat’s clinical application is hindered by its low aqueous solubility and high lipophilicity, which limit its bioavailability [1]. Recent studies have explored various strategies to enhance its solubility and release profile, including nanosuspensions and polymer-based systems [2]. However, these approaches often involve complex manufacturing processes and higher costs.

A research gap lies in developing a simpler, cost-effective method to achieve extended-release (ER) formulations, improving patient compliance and therapeutic outcomes. Activated charcoal, known for its high surface area and adsorption capacity, offers a promising solution as an adsorbent in ER formulations. Its use could provide a controlled release mechanism while lowering production costs [3].

FEB is a critical medication for managing hyperuricemia in patients with gout [4], but its clinical use is limited by low aqueous solubility and high lipophilicity [5]. Improved formulations are necessary to enhance its bioavailability and patient compliance. To overcome these challenges, this study aimed to develop a cost-effective, extended-release formulation of febuxostat using activated charcoal as an adsorbent. Charcoal (activated carbon) is a strong and efficient drug and toxic compound binder that could be used in various drug delivery systems [6,7]. Activated charcoal is widely used in pharmaceuticals because of its distinct physicochemical qualities, such as its high surface area and porosity, which allow it to function as an effective adsorbent. This makes it valuable for use as a diluent in drug formulations, where it can attenuate the absorption of specific pharmaceuticals and toxins in the gastrointestinal tract, limiting their bioavailability and possible toxicity [8,9]. Methods used in this research involved creating eight formulations with varying febuxostat-to-charcoal ratios. Key techniques included Fourier-transform infrared spectroscopy (FTIR) and differential scanning calorimetry (DSC) to analyze physical interactions. In vitro dissolution tests and in vivo pharmacokinetic studies in rats were conducted to assess drug release and bioavailability. This study’s impact is that its novel formulation offers a promising solution for controlled febuxostat release, reducing dosing frequency, improving patient compliance, and lowering production costs. Its development marks a significant advancement over existing formulations, potentially leading to better clinical outcomes for patients with gout.

FEB is a class II Biopharmaceutical Classification System (BCS). It is a non-purine selective inhibitor of xanthine oxidase, which is responsible for the oxidation of hypoxanthine to xanthine and, subsequently, uric acid. The production of uric acid could be controlled by inhibiting this enzyme, a critical step in the purine catabolic pathway [10,11]. FEB was approved in February 2009 by the FDA for Takeda pharmaceuticals [12].

**Figure 1:**
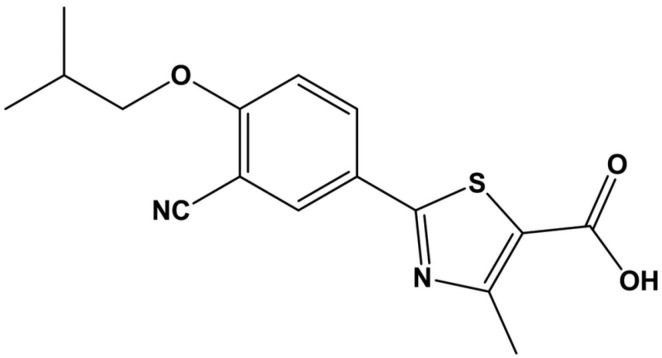
Chemical structure of FEB [3].

Chemically, FEB is 2-(3-cyano-4-isobutoxyphenyl)-4-methyl-5-thiazolecarboxylic acid [13]. The structure is shown in Figure 1. Its structure consists of a thiazole ring, a cyano group, and a carboxylic acid functional group, contributing to its distinct pharmacological properties [14]. Physicochemically, FEB is characterized by low aqueous solubility and high lipophilicity, challenging its formulation into effective oral dosage forms [15].

ER tablets are designed to release their active ingredient at a predetermined rate to achieve sustained therapeutic levels over an extended period [16]. There are several types of ER tablets, including matrix tablets, reservoir systems, osmotic pumps, and gastroretentive systems [17]. Each type has a design that ensures the active ingredient’s slow or controlled release. Matrix tablets typically employ hydrophilic or hydrophobic polymers that control drug release through diffusion and erosion mechanisms. Reservoir systems, on the other hand, use a core containing the drug, surrounded by a rate-controlling membrane that regulates the drug’s release. Osmotic pump systems utilize osmotic pressure to deliver the drug at a controlled rate, while gastroretentive systems are designed to prolong the drug’s residence time in the stomach, extending its release duration [18,19,20,21].

Several studies have investigated different types of controlled release systems of oral FEB to enhance its pharmacokinetic profile and patient compliance. For instance, Tayyeb et al. developed a FEB-loaded chitosan polymeric nanoparticle system, demonstrating improved solubility and prolonged drug release compared to conventional formulations. In this system, FEB was released by diffusion and biodegradation of chitosan over the specified period [22]. Nanosuspension was another approach to enhance the oral bioavailability of FEB. The system showed improved solubility, dissolution rate, and bioavailability in the Wistar rat model [23]. Rathi et al. developed a gastroprotective floating tablet of FEB using Polyox N-60K and Carbopol 934 P in combination, which was optimized, as it slowed drug release up to 12 h. The system showed a prolonged release mechanism depending on the swelling mechanism [24]. Additionally, research by Sohn and Choi 2023 explored the stabilization of dispersion using the effect of dicalcium phosphate dehydration on chitosan nanoparticles to improve in vitro release and permeability of FEB [25].

Recent advancements in pharmaceutical technology have led to the development of novel carbon-based materials for drug delivery systems. These materials, such as graphene oxide and carbon nanotubes, offer unique properties, including high surface area, mechanical strength, and biocompatibility, making them suitable for extended-release formulations [26]. However, the high cost of such systems might be an obstacle at the industrial level.

This study aims to design a cost-effective, eco-friendly, extended-release FEB tablet using adsorption on charcoal phenomena to control FEB release. Only one type of diluent and one disintegrant are aimed to be used to produce the tablet. The tablet’s in vitro and in vivo performance was used to evaluate this design.

This approach might be beneficial in decreasing the cost of ER tablet production in terms of the number and price of the excipients used and in terms of its ease of production and evaluation. This study aimed to address these challenges by developing an ER febuxostat tablet utilizing activated charcoal. We hypothesized that incorporating charcoal would enhance the drug’s release profile and bioavailability. By systematically evaluating various formulations, we sought to identify an optimal febuxostat-to-charcoal ratio for achieving sustained therapeutic levels.

## 2. Results

### 2.1. Method of Analysis by HPLC

**Figure 2:**
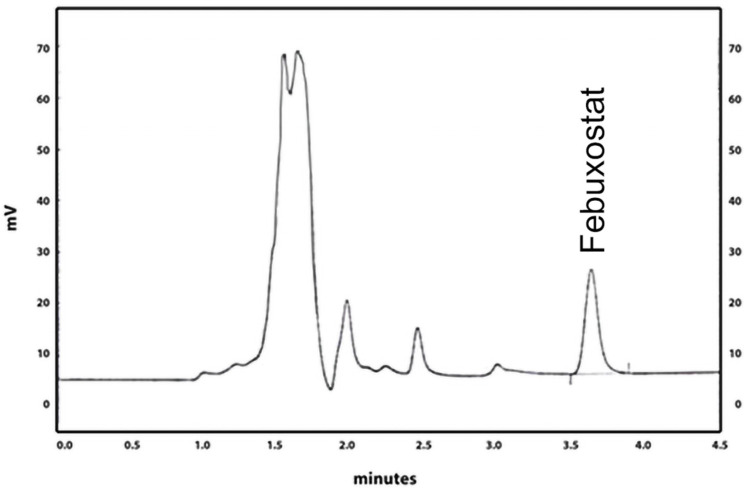
A chromatogram of FEB shows a retention time (RT) of 3.7 min.

**Figure 3:**
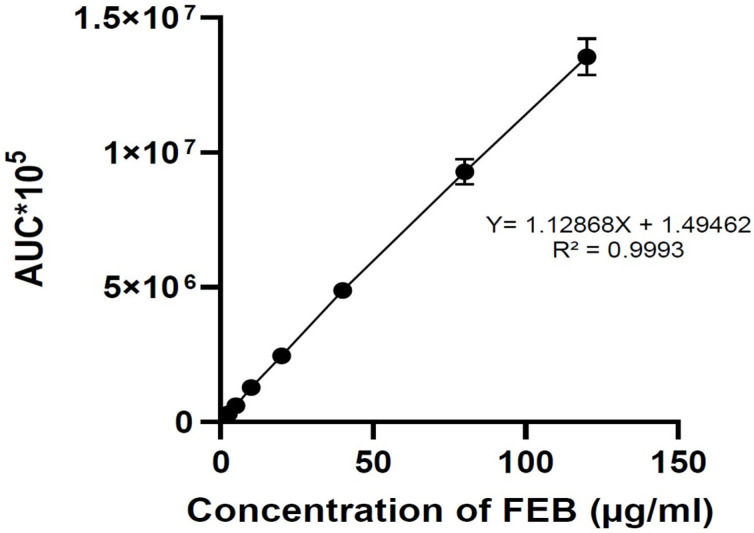
The calibration curve and linearity data of FEB show an R^2^ of 0.9993 and a linearity equation.

An FEB chromatogram is shown in Figure 2 with a retention time (RT) of 3.7 min. The linearity of the method measured (R2) equals 0.9993 (Figure 3). The precision of the technique produced RSD 0.4–1.1 for the three QC values and an accuracy of 98.2–102.6%.

### 2.2. Evaluation of the FEB-CHR Mixture

**Figure 4:**
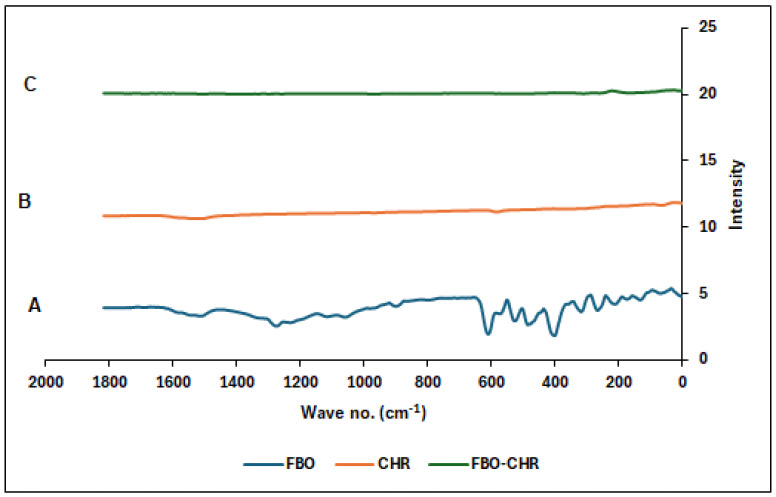
FTIR spectrum of (A): FEB, (B): CHR, and (C): FEB-CHR adsorbate powder showing the absence of the major peaks of the drug in the powder mixture prepared by the trituration method.

**Figure 5:**
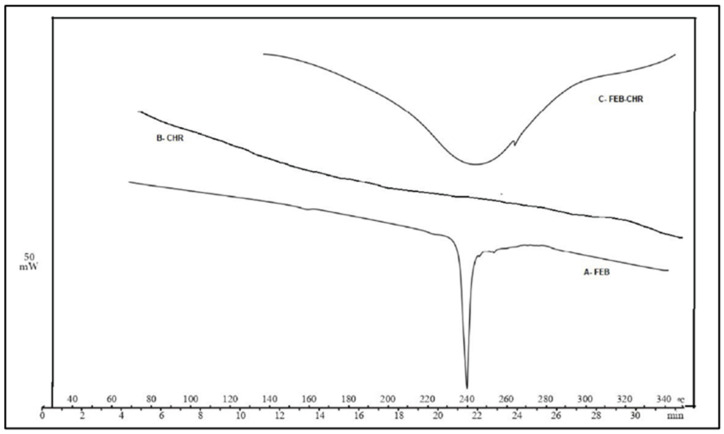
Thermogram of FEB (A) showing its melting point at 240 °C. With CHR (B), no peak is observed due to the high melting point, and FEB-CHR (C) adsorbate powder shows a wide broad peak indicative of a physical interaction.

FTIR and DSC evaluated the powder result of the adsorption process. The FTIR results are shown in Figure 4 and Figure 5, respectively.

The particle size analysis showed a uniform distribution of the FEB-CHR powder particles; the average particle size was 830 ± 254 nm, and the dispersity (Ð.) was 0.08.

**Figure 6:**
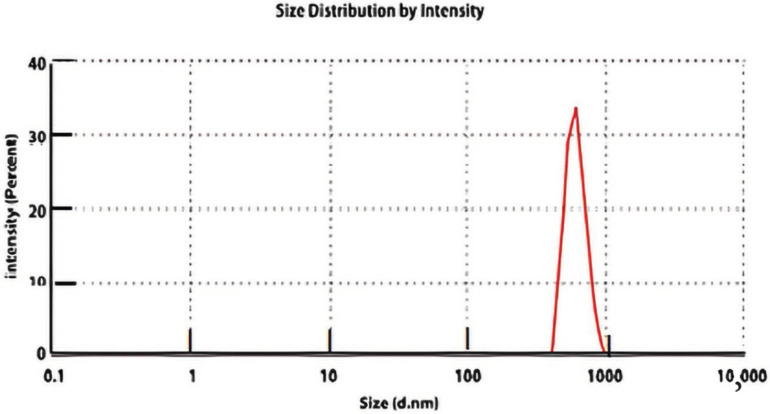
The particle size distribution measurement of FEB-CHR powder shows a single peak with an average particle size of 830 ± 254 nm.

Figure 5 shows the thermogram of FEB, and Figure 6 shows the particle size distribution.

For the adsorption density (AD), results of adsorption density showed that the data fit pseudo-first-order kinetics (0.96), with an adsorption constant of 0.015 mmol/h.

### 2.3. Flowability and Compressibility

The compressibility of the powder blends of the formulations was evaluated, and the results are shown in Table 1.

### 2.4. Evaluation of the Tablets

The tablets with an FEB-to-charcoal ratio of 1:1 and higher showed a black color, which might not be attractive, and the tablets with lower ratios of charcoal looked almost grey. The tablets were intended to be coated with an attractive color. But for this trial, they were evaluated as they were after compression. Hardness, friability, and disintegration results are shown in Table 2.

### 2.5. Dissolution and Drug Release

**Figure 7:**
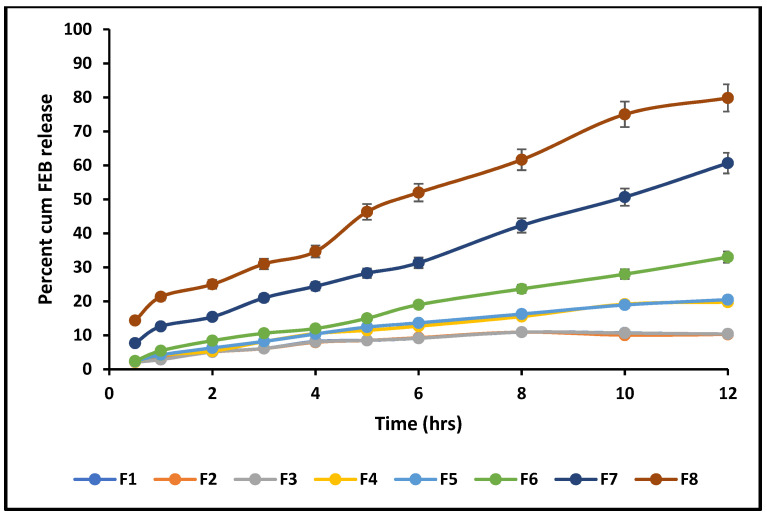
Release profile of the prepared formulations at pH 6.8 using dissolution apparatus II, 50 rpm, and 37 °C. Results show the highest percent of release from F8, which contained FEB:CHR in a ratio of 1:0.25.

Figure 7 shows the results of the release study at pH 6.8. The cumulative drug release ranged between (20 and 80%) of FEB from the tablets. At pH 1.2, the total drug release was slightly lower than at pH 6.8 (20–75%) for all formulations.

The release data of the prepared formulation were fitted on several release models: the zero-order release model, the first-order release model, the Peppas model, the Higuchi model, and the Hixson–Crowell model. The data correlation was expressed by R2 and is tabulated in Table 3 below.

### 2.6. In Vivo Pharmacokinetic Study

**Figure 8:**
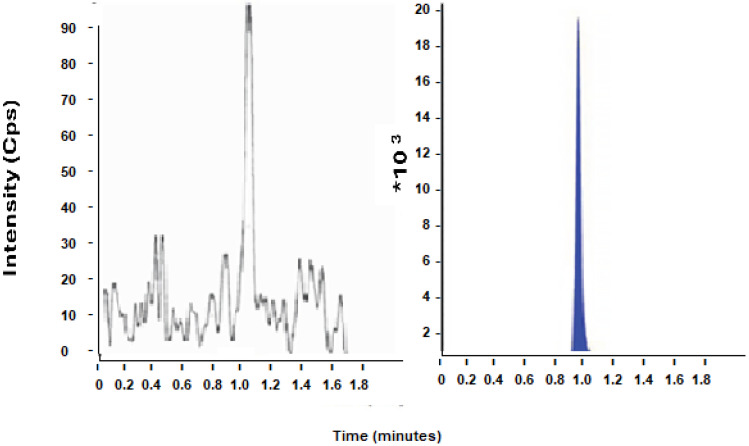
Chromatogram of FEB in rat plasma (from linearity test). RT was equal to 1 min.

The LC/MS-MS analysis showed the following peak extracted from the rat’s plasma. RT was equal to 1 min, making this method quick and economical, as shown in Figure 8.

Linearity of the method was achieved between 1 and 200 ng/mL, precision and accuracy of three QC levels was between 2 and 6 expressed by RSD 0.6–1.0, and recovery was 97–105 %.

**Figure 9:**
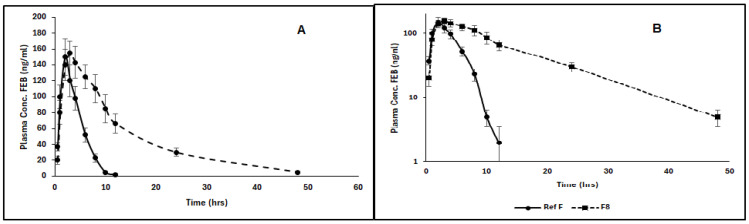
Plasma level–time profile of FEB in rat plasma of F8 and the reference formula. (**A**) regular scale and (**B**) semilog scale.

The plasma level–time profile of the newly proposed formulation (F8) is shown in Figure 9. The pharmacokinetic parameters are shown in Table 4.

## 3. Discussion

FEB is class II BCS medication. Several attempts were made to improve oral bioavailability in addition to controlled release preparation to increase patient compliance. The CR preparation used several approaches to control the release of the API up to 12 or 24 h from tablets with good quality and reasonable cost. Problems like dose dumping and burst effects usually represent challenges in this aspect.

The study focuses on the extended-release febuxostat tablets’ effectiveness and reliability in maintaining sustained therapeutic levels, achieving long-release profiles, and maintaining effective drug concentrations. The 12 h test duration aligns with these objectives, minimizing the need for frequent dosing. The optimal formulation demonstrated a consistent release rate over 12 h, which is desirable for maintaining effective drug concentrations. The 12 h time frame is commonly used in pharmacokinetic studies to assess the performance of extended-release formulations, supporting regulatory compliance. The choice may be influenced by prior studies that established 12 h as an effective duration for evaluating similar drug formulations [27,28]. The study aims to demonstrate the efficacy and reliability of the extended-release formulation in improving patient compliance and therapeutic outcomes.

This work first used an HPLC method to measure FEB, the target API. The technique used an HPLC system with a UV detector on 320 nm and a mobile phase consisting of 40% phosphate buffer of pH 3 and 60% acetonitrile. The method proved linearity of 0.9996 in the 2.5–120 µg/mL range. The precision of the three QC samples ranged between 0.4 and 1.1 as the value of RSD and showed an accuracy of 96–103%. Recovery of the method was 96%. Analysis of placebo tablets showed no peak of FEB at the same chromatographic conditions.

The formulation design was based on creating a powder mixture of FEB and CHR by adsorption of FEB on the CHR using the trituration method. This phenomenon was used as the retarding force to release FEB from the powder in a controlled manner. CHR is an adsorbent used in many toxicity cases, so the amount of CHR used relative to the amount of FEB was the major investigation variable in this work. Eight formulations were prepared with different weight ratios of FEB and CHR in the 1:2 to 1–0.25 of FEB:CHR. The prepared adsorption powder was achieved by trituration using mortar and pestle. CHR is an inorganic amorphous material. It is insoluble in water, and organic solvent-making methods involve very difficult solvents with expected weak performance to maintain the low cost and eco-friendly method. A dry process would be safe and cost-effective. The powder was dry and brittle, which was expected to create a problem in the compression; for this reason, a wet granulation method was applied to improve the powder characteristics.

FTIR, DSC, PS, and AD evaluated the FEB-CHR powder. The FTIR spectrum of FEB alone (Figure 4-A) showed the major peaks of FEB, which were C=O, C-O, O-H stretching, and O-H bending, belonging to the carboxyl group 1760, 1320, 3300–2500, and 1440–1395 cm^−1^, respectively, and peaks at 2228.03 cm^−1^ (C=C stretching), 1014 cm^−1^ (C=N-stretching), and 758 cm^−1^ (≡C-O-stretching), in addition to the C-H at 2956 cm^−1^.

CHR consists of carbon atoms, but activated charcoal has demonstrated binding of carbon atoms to hydrogen and oxygen. Figure 4-B showed two distinctive peaks at 1625 cm^−1^, which might belong to aromatic C=C ring stretching [29] as described elsewhere, and a broad band at 3400 cm^−1,^ formed by O-H stretching [30].

The powder formed by trituration of FEB and CHR (1:1 ratio) (Figure 4-C) showed a spectrum that was close to that of CHR alone, indicating hidden groups of FEB in the CHR, with solid evidence of physical binding between the two compounds.

The results of the DSC analysis are shown in Figure 5. An FEB thermogram (A) showed a sharp endothermic peak at 240 °C representing the melting point of crystalline FEB [31]. At the same time, B represents the thermogram of charcoal, which does not melt in the temperature range of the test. Charcoal melts at 1130–1300 °C. Figure 5-C is the thermogram of the mixture of FEB-CHR formed by the adsorption process. It shows a broad peak in the range of 220–280 °C instead of the sharp melting-point peak. Such broad peaks have often been reported as a sign of physical interaction between an API and an adsorbent or polymer [32]. This further supports the adsorption process of FEB on the surface of CHR.

The company supplied the activated CHR as a powder for the PS analysis. However, the trituration process with FEB might result in a change in the PS. The particle size might play a role in the release of the API from the surface of the powder. Results showed that the PS was 820 ± 225 nm, which is less than one micron and offered a good surface area for the FEB to be adsorbed even when the amount of CHR used in the formula was less than the API. This is another advantage. The balance between the adsorption force and the surface that will be wetted in the release test might determine the pattern of FEB release.

The extended-release formulation of FEB using activated charcoal has been found to enhance its release profile and overall bioavailability. The adsorption characteristics of activated charcoal, such as its high surface area and porous structure, facilitate the adsorption of FEB, enhancing its dissolution [33]. This creates a reservoir effect, where the drug is gradually released as it desorbs from the charcoal surface. The optimal formulation (F8) showed a consistent 12 h zero-order release pattern, maintaining steady plasma concentrations and minimizing side effects [34,35]. The release profile was assessed at different pH levels, with formulations performing better at pH 6.8 compared to pH 1.2 [11]. This is due to the ionization state of the drug, which influences its interaction with the adsorbent. The FEB-to-charcoal ratio significantly affected the release rate, with formulations with a higher proportion of charcoal exhibiting enhanced drug release rates [36]. This highlights the importance of optimizing excipient ratios to achieve desired release profiles. In vivo pharmacokinetic studies corroborated these findings, showing that the F8 formulation provided a higher AUC than the reference immediate-release formulation. This correlation between in vitro release and in vivo bioavailability supports the effectiveness of the developed formulation in achieving sustained therapeutic levels [37]. Future studies should explore this formulation’s long-term stability and clinical implications to understand its potential benefits fully.

The adsorption density measurement shows how the adsorbate could adapt to the API. Using the equal weight of CHR and FEB, 0.015 mmol/h was obtained, which could be relatively high, supporting the CHR’s high absorbing capacity.

The powder blends of the formulations showed good flowability, except F1, which showed “fair” flowability. This might be attributed to this formula’s high amount of charcoal. It could also be related to the granulation process and its technique. Table 2 shows the physical characterization of tablets of all formulas. The hardness of the tablets showed increasing values with a decreasing percentage of CHR, which means that even with the granulation process, the amount of CHR produces brittle granules, which, in turn, give rise to reduced hardness. Friability results were consistent with hardness, where a lower amount of CHR produced less-friable tablets. Also, the disintegration time increased with the decreased CHR amount, but all results were accepted. Since the design was put in place to release the API from the surface of CHR, these parameters were all aimed to fit the IR tablet preparations.

Drug release from the eight formulations was measured at pH 6.8. The total drug release after 12 h was compared statistically. F8 produced the highest amount of release, followed by F7. F1–F6 produced a limited amount, not exceeding 33% and achieving 67% of the total drug release of the F8 Hixson–Crowell model, with a total drug release of 80%. This might indicate a promising result for the proposed in vivo design. CHR is known as a potent adsorbent, and the drug release from its surface represented the biggest challenge. That is why an in vivo bioavailability study was necessary. The bulking agent used was Avicel PH 102, which possesses good wetting property that might enhance the dispersion of the FEB-CHR powder. The interaction of FEB with water and the energy balance with the forces of adsorption created by the surface interaction of FEB with CHR would be the major factor affecting FEB release. Statistically, the effect of Avicel on the release was not considered a test variable. However, increasing Avicel corresponding to the decreasing CHR might have improved the powder’s dispersion, contributing to a more uniform FEB release. F1, F2, F3, and F4, which contained FEB:CHR ratios of 1:2 to 1:1.25, showed a non-significant difference in total drug release over 12 h (*p* > 0.05), while the ratio of FEB:CHR of 1:1 up to 1:0.25 increased the total drug release.

The FTIR and DSC results also supported the physical interaction of FEB at the surface of the CHR. Furthermore, the particle size measurement showed particles of an average size of less than 1 µm, which provides a large surface area that helped the distribution of FEB on the surface. This might contribute to the uniform and consistent release of FEB.

Data on drug release were fitted on several release models: the zero-order release model, the first-order release model, the Peppas model, the Higuchi model, and the Hixson–Crowell model. These models form the pattern of the release profile as a function of time rather than describing the release process in terms of a related differential equation. Mathematical models describing drug release kinetics from controlled-release pharmaceutical dosage forms can be categorized into two groups. The first group is concerned with the release of the drug, which is defined by the mechanism of breakdown of the delivery system, while the second group is characterized by the rate of release as the mechanism of drug transport through the matrix forming the controlled-release system. Since our proposed system depends on the de-binding of the adsorbed drug on the surface of the CHR particles, it cannot exactly be considered as a “diffusion process”. This might explain why F8, chosen as the promising formula, correlated the highest to the Hixson–Crowell model, which describes powder dissolution from spherical particles. This supports our hypothesis of controlling the release of CHR by a dissolution process rather than by diffusion through a matrix. It also correlated highly with the “zero-order” release model, which ensures an almost constant release over the test period.

FEB is a weak acid that has lower solubility in acidic media. However, the release of FEB seems pH-independent, as shown by preliminary experiments in acidic media, where no burst effect was noticed.

F8, in the form of a reference tablet containing FEB with additives such as IR tablets, was provided to the animals.

The LC-MS/MS method was reliable and fast, and the validation parameters were all within the specifications of the guideline. An analysis of samples showed that the plasma concentration over the time of the experiment was within the range of the linearity test.

PKP analysis showed that the Cmax from F8 and the reference formula were statistically non-significant (150 ± 25 vs. 155 ± 35 ng/mL), which means that the initial rate of absorption was the same in both and that FEB from F8 started to be released and reached the same Cmax of the IR formula. This is also supported by the non-significant difference in the Tmax (2.00 ± 1.0 vs. 3.00 ± 0.5 h). However, the AUC0-t from the F8 was significantly higher than that of the reference formula (665.5 ± 120 ng.h/mL vs. 1134.5 ± 200 mg.h/mL with *p* < 0.001). This means that a higher amount of FEB was released from F8, and the plasma concentrations were higher than those in the reference formula. All these results illustrated that F8 provided FEB release and absorption over a long period compared to the amount absorbed from the reference formula.

The results showed promising results for designing FEB adsorbed on CHR as a simple, cost-effective method to control FEB release, thereby offering a potential substitute for multi-dosing IR tablets.

### 3.1. Pharmacokinetic Parameter Comparison

The study compared the ER formulation of FEB with an immediate-release (IR) reference. Key pharmacokinetic parameters were analyzed:

1. Cmax (maximum concentration):

The ER formulation showed a slightly lower Cmax compared to the IR reference. This indicates a more gradual release and absorption of the drug, reducing peak plasma levels and potentially minimizing side effects.

2. Tmax (time to reach Cmax):

The ER formulation had a longer Tmax, reflecting the slower release and absorption rate. This extended Tmax is desirable for maintaining therapeutic levels over an extended period.

3. AUC (area under the curve):

The ER formulation demonstrated a higher AUC, indicating greater overall drug exposure over time. This suggests improved bioavailability compared to the IR formulation.

A comparison of different formulations was carried out. The F8 formula demonstrated significantly higher AUC than the reference formulation (*p* < 0.001). In terms of drug release, the F8 formula had a substantially higher release profile than F1–F6 (significance was tested using appropriate statistical tests (e.g., ANOVA), with *p*-values < 0.05 indicating significance). There was no significant difference between the F8 formula and the reference when comparing pharmacokinetic parameters such as Cmax and Tmax. However, the AUC0–12 and AUC total of the F8 formula were significantly higher (*p* < 0.001). In conclusion, the F8 formulation outperformed other formulations regarding drug release and bioavailability, as evidenced by significant statistical differences in AUC. The findings support using activated charcoal in extended-release formulations to improve febuxostat bioavailability.

### 3.2. Consistency and Correlations between In Vitro and In Vivo Outcomes

In vitro drug release: the F8 formulation demonstrated the highest release profile in vitro, with an 80% cumulative release, while in terms of in vivo pharmacokinetics, the F8 formula showed significantly higher AUC than the reference, indicating improved bioavailability.

The in vitro release profile of the F8 formula was consistent with the in vivo results, as both showed increased drug availability and absorption over time.

The discrepancies can be seen in Cmax and Tmax. Despite the F8 formula’s superior release in vitro, there were no significant differences in Cmax and Tmax in vivo.

However, in vitro data indicated rapid release, and in vivo results revealed a similar initial absorption rate to the reference, possibly due to physiological factors influencing drug release and absorption.

Overall, the in vitro and in vivo results for the F8 formulation were highly correlated, particularly regarding bioavailability (AUC). However, minor variations in initial absorption rates highlight the difficulty of translating in vitro results directly to in vivo outcomes.

Regarding physicochemical interactions between FEB and charcoal, FEB is adsorbed onto activated charcoal, which increases its surface area and may enhance dissolution. This adsorption increases the surface area for dissolution, thereby increasing the drug’s release rate. The interaction promotes a sustained release profile as the drug gradually desorbs from the charcoal surface. Improved dissolution results in better absorption in vivo, as evidenced by higher AUC values for the optimal formulation (F8). The controlled release helps to maintain consistent plasma levels, which improves therapeutic efficacy and patient compliance. Overall, the interactions between FEB and charcoal improve dissolution and absorption, making the formulation better suited for extended-release applications.

This study presents a novel approach to drug release using activated charcoal. This approach is a cost-effective and innovative method compared to traditional polymer-based systems. The study shows a consistent 12 h zero-order release pattern, improved bioavailability, and enhanced patient compliance. The controlled release reduces dosing frequency, making it more effective than immediate-release formulations. The use of charcoal also reduces production costs compared to complex nanosuspensions and polymer systems used in previous studies. The methodology includes comprehensive FTIR and DSC analysis to understand the formulation’s behavior. This study offers significant advancements in developing cost-effective, extended-release FEB tablets with novel contributions in enhancing dissolution and bioavailability. The findings present a promising alternative to ER formulations, potentially leading to better therapeutic outcomes.

The formulation of the new drug shows improved bioavailability and a 12 h zero-order release pattern, potentially improving patient outcomes. It is cost-effective due to activated charcoal, a cost-effective adsorbent, and is produced through a simplified manufacturing process. This makes it more accessible for large-scale production and reduces time and resource investment. The formulation offers a promising alternative, balancing efficacy with cost and production advantages. Its improved release and absorption make it comparable or superior to existing formulations; thus, it is more affordable and easier to produce.

The study has limitations such as a small sample size, potential variability in activated charcoal sources, and a need for long-term stability data. The sample size may need to capture these factors adequately, and the absence of long-term stability studies may impact the formulation’s shelf life and effectiveness over time. Addressing these limitations in future research may provide a more complete picture of the formulation’s reliability and applicability.

This study has several limitations, including the studied drug’s effectiveness in treating gastrointestinal issues. The study was carried out on rats, which may not accurately represent human physiology. The short-term evaluation concentrated on short-term pharmacokinetic data without considering long-term effects or safety. The study also needed to thoroughly investigate the precise mechanisms of drug release and interaction with charcoal. The drug release was tested under limited pH conditions, which did not account for the full range of variability in human gastrointestinal environments. The study also needed to address potential scale-up issues in industrial production. This limitation could be addressed in future studies by several strategies such as improving the reliability and generalizability of results in the formulation of a drug. These could include larger sample sizes, considering clinical trials with human participants, examining the variability in activated charcoal sources, standardizing the charcoal properties used in the formulation process, conducting extended stability testing to evaluate the formulation’s shelf life under different conditions, and assessing the impact of storage on drug release and efficacy over time.

## 4. Materials and Methods

### 4.1. Materials

FEB (Sigma Aldrich, Saint Louis, MI, USA, purity 99.8%), activated charcoal (Thermo Fischer, GmbH, Berlin, Germany, purity > 98%, humidity < 5%), and all solvents were HPLC grade from Analar, London, UK. Tablet excipients were kindly gifted from Dar Aldwa Pharmaceuticals, Jordan.

### 4.2. HPLC Method of Analysis of FEB

The method of analysis was adapted from Rao et al. [38]. The high-performance liquid chromatography (HPLC) system (Thermo Electron Corporation, San Jose, CA, USA) included a pump (LC Pump plus, Thermo Finnigan, San Jose, CA, USA), an intelligent, sensitive liquid chromatography pump with an auto-sampler programmed at 10–20 µL capacity per injection applied in the system. A UV–vis detector was used Thermo Finnigan Surveyor UV/Vis PLUS Detector (Surveyor PDA Plus). This model operated and was fixed at a wavelength of 320 nm. The column used was Thermo Hypersil type-3-BDS-C18 (200 mm × 4.6 mm, 5.0 µ, Thermo Fisher Inc., Waltham, MA, USA).

A 1 mg/mL stock solution was prepared by dissolving 10 mg FEB in the mobile phase consisting of 40 mL phosphate buffer of pH 3 and 60 mL acetonitrile. To prepare a 40% phosphate buffer at pH 3 and a 60% acetonitrile solution, we used sodium phosphate (monobasic (NaH_2_PO_4_) and dibasic (Na_2_HPO_4_)), distilled water, and a pH meter or pH indicator strips. For the 40% phosphate buffer, we dissolved phosphate salts in distilled water and adjusted the pH using a pH meter or HCl. We transferred the solution to a volumetric flask and diluted it with distilled water to reach the desired pH. For the 60% acetonitrile solution, we measured 600 mL of acetonitrile and diluted it with water to obtain the total volume. We stored both solutions in labeled containers, with the phosphate buffer stored at room temperature and the acetonitrile stored in a cool, dark place. We used appropriate personal protective equipment (PPE) when handling chemicals. Both solutions should be stored in labeled containers and maintained at room temperature. Serial dilutions generated a calibration curve in the 2.5–120 µg/mL range. For precision and accuracy, 3 QC samples were used: 5 µg/mL, 50 µg/mL, and 100 µg/mL. Regarding recovery, this test was applied using a powder mixture containing all the additives used in the formulation.

Results were all evaluated according to the ICH guideline of pharmaceutical analysis [39].

### 4.3. Preparation of the Tablets

Eight formulations were prepared using different ratios of FEB to charcoal (CHR). Preliminary studies showed that a high ratio of charcoal resulted in a depleted release of FEB due to the known high capacity of adsorption of charcoal. So, the formulation is based on trials using the optimum amount of charcoal to provide the drug with a prolonged release.

The trituration method was used to prepare the FEB-CHR powder due to its advantage of avoiding moisture and solvents, because this method is eco-friendly, and because this method is cost-effective. In all formulations, 40 g of FEB was placed in a large porcelain mortar. Then, the specified amount of CHR (ranging between 10 and 80 g) was added, and a one-direction trituration process was performed manually using the pestle and carefully including all the powder material. The mixture was then transferred to a cube mixer (Erweka AR 403, 400 × 440 × 450 mm R&D scale, Langen, Germany). Then, 5 g of sodium starch glycolate (SSG), as a disintegrant, and Avicel (73 to 143 g, as a bulking agent) were added together while the mixer rotated at 100 rpm. Finally, after 30 min, the powder was transferred to the granulator to perform the wet granulation process suggested after direct compression trials, which produced very low-quality brittle tablets. All experiments were performed in the Industrial Pharmacy lab under controlled conditions of 26 ± 2 °C and 10% RH.

A wet granulation process was performed using a distilled water–ethanol mixture (1:1) as granulating liquid using a granulator (FGS granulator, Erweka, Heusenstamm, Germany); then, the granules were dried and sieved using a no. 35 sieve manually. Subsequently, lubricant was added, and 5–7 min of further mixing was applied before compression. This process was performed using the same cube mixer.

A compression process was performed using the Bhagwati Mini Press, Ambala, India, R & D scale. An amount of 500 tablets of each formula were prepared. Table 5 shows the composition of the formulations.

The aim was to rely on the interaction between CHR and FEB as a retarding force that might be able to control the drug release over a long period. This would save many steps and costly materials usually used to optimize controlled-release formulations.

### 4.4. Evaluation of the FEB-CHR Mixture

#### 4.4.1. Fourier-Transform Infrared (FTIR) Spectroscopy

FTIR was performed on CHR and FEB, respectively, and on the mixture after the trituration process, using a Shimadzu Europa-Model: LC-2030C PLUS (Shimadzu, Kyoto, Japan). Samples were scanned 400 to 4000 cm^−1^, and the spectrum was recorded and examined.

#### 4.4.2. Differential Scanning Calorimetry (DSC)

DSC was performed on a Mettler Toledo 1 STAR System, San Sebastian, Spain. CHR, FEB, and their mixture were subjected to this test. The temperature applied was from 4 °C to 400 °C. Thermograms were recorded and examined.

#### 4.4.3. Particle Size Measurement

The average particle size of FEB-CHR powder was measured with a Malvern Zetasizer, Malvern Panalytical, Worcestershire, UK. A 1 mg sample was suspended in 5 mL of deionized water and shaken vigorously in a vortex. An amount of 100 µL of this suspension was diluted to 1 mL by deionized water and read three times. Average ± SD and Ð. were recorded.

#### 4.4.4. Adsorption Density

A batch adsorption experiment was performed as described by Chang et al. 2015 [40]. The equilibrium adsorption was conducted in a batch experiment at a stirring speed of 140 rpm for 72  h and at 25 °C. The initial concentrations of FEB and CHR were both kept constant at 10 mg/L, and the particle size was measured in the previous experiment. Supernatants were obtained by filtration using fiberglass membrane (Millex HA 0.45 μm filter, Millex Millipore, Barcelona, Spain) and stored at room temperature before analysis for residual concentration of FEB.

The adsorption density was determined from the initial and residual concentrations of the adsorbate according to the following equation: [41,42]

qt = v/m (Co − Ct)(1)

where qt is the adsorption density at time t (mmol/g); v is the volume of solution (L); Co is the initial solute concentration (mmol/L); Ct is the solute concentration at time t (mmol/L); and m is the amount of CHR used (g).

### 4.5. Evaluation of Compressibility of the Granules

Since wet granulation was applied to the formulation powder, the granules were evaluated for flowability using the angle of repose as a parameter according to the USP. Also, Carr’s Index and Hausner’s ratio were calculated after measuring bulk and tapped density [43].

### 4.6. Evaluation of the Prepared Tablets

Weight uniformity, hardness, friability, and disintegration time were all evaluated according to the USP 32 for the tablet dosage form.

A dissolution test was performed using USP apparatus type II (Paddle Apparatus, Erweka® DT600 dissolution test system (Erweka GmbH, Germany)), 50 rpm, and 900 mL dissolution media. The test was conducted at two pH values: 1.2 (0.1NHCl) and 6.8 (phosphate buffer). The test was performed on all the formulations for 12 h.

### 4.7. In Vivo Study and Measurement of Bioavailability

#### 4.7.1. LC-MS/MS Method of Analysis of FEB in Rats’ Plasma

In a previous study, an LC/MS/MS analysis method for FEB in human plasma was developed and validated for bioequivalence. A slight modification was made to apply the same process in this study. The validation parameters (linearity, precision, accuracy, and recovery) were all repeated in this study.

The mobile phase used was composed of 70% methanol with 30% of a mixture of 30 mM ammonium acetate and 0.3 mM formic acid and was eluted isocratically with a flow rate of 1 mL/min through an ACE™ C18 (50 × 2.0 mm, 5 μm) column for LC-MS/MS. The injection volume was 2 μL. The MS parameters were adjusted for FEB and Sitagliptin (internal standard) analysis in ESI negative mode. Samples were prepared using the protein precipitation method with acetonitrile.

Linearity of the method was measured in the range of 5 ng/mL–300 ng/mL, and the precision and accuracy used three QC samples (QClow 5 ng/mL, QCMid 80 ng/mL, and QCHigh 250 ng/mL) by measuring six replicates and measuring the RSD. The acceptance criteria of all tests were evaluated according to the ICH guideline [39].

#### 4.7.2. Model Tablet Preparation

A simple direct compression method was performed to prepare the model tablets administered to the rats. FEB, avicel, SSG, and Mg stearate were used to prepare tablets weighing 25 mg and containing 5 mg FEB with the same percentage of disintegrant and lubricant. The controlled-release tablets were compressed using the powder blend of the selected formula, equivalent to 5 mg FEB with a final weight of 25 mg.

#### 4.7.3. Measurement of Pharmacokinetic Parameters

Two groups of Sprague Dawley rats were used in this study. The rats’ average weight was 230 g ± 10 g, they were 8–9 weeks of age, and they were all males. G1, composed of six rats, was administered the reference tablets, and G2 was administered the test tablets of the selected formula containing 5 mg FEB. The animals were climatized in an animal house with 12 h cycles of light and darkness and free access to food and water for 3 days before the experiment. Fasting was achieved overnight (10 h), and dosing was administered at 8:00 a.m.

The tablets were crushed into 3–4 parts with 2 mL of water and administered via gastric lavage to the animals. Samples were obtained for 48 h at the following time points: 0.5, 1, 2, 3, 4, 8, 12, 24, 36, and 48 h. Samples of 300 µL were withdrawn each time from the tail after warming with a warm pad with a massage. Samples were drawn in heparinized tubes, centrifugated, and stored at −70 °C until analysis.

The plasma level–time profile was then constructed by plotting the concentration of FEB vs. time for all rats, and the data were treated by WinNonlin^®^ 8.3 for non-compartmental analysis. Cmax, Tmax, and AUC_0-t_ were calculated to evaluate the in vivo performance of the selected formula.

#### 4.7.4. Statistical Analysis

Statistical analysis for the in vitro study was performed using SPSS version 26. A multivariant ANOVA test was conducted in the release study. A comparison of definite values was performed using a *t*-test. All of these tests used CI 0.05%. The statistical analysis for the pharmacokinetic analysis is included in the Phoenix WinNonlin® software (Version 8.3, Certara USA, Inc.)

## 5. Conclusions

This study successfully developed an extended-release FEB tablet based on activated charcoal, optimizing the formulation to achieve a consistent 12 h zero-order release profile. The optimal formulation, the F8 formula, showed significantly improved bioavailability, as evidenced by higher AUC values than traditional formulations. This novel approach improves patient compliance while lowering production costs, indicating a promising solution for effective and accessible gout management. The findings emphasize the potential benefits of using low-cost materials to achieve sustained drug release and improve therapeutic outcomes.

## Figures and Tables

**Table 1:** Results of flowability and compressibility of the prepared formulations.

Formula Code	Ratio of FEB:CHR	The Angle of Repose (θ)	Carr’s Index	Hausner Ratio	Flowability Description
F1	1:2	36 ± 1.0	18.2 ± 1.0	1.18 ± 0.05	fair
F2	1:1.75	35 ± 1.5	17.0 ± 0.5	1.15 ± 0.03	good
F3	1:1.5	34 ± 2.0	13.0 ± 0.5	1.13 ± 0.02	good
F4	1:1.25	33 ± 1.0	13.0 ±0.9	1.12 ± 0.05	good
F5	1:1	33 ± 0.75	12.8 ± 1.0	1.11 ± 0.04	good
F6	1:0.75	32 ± 1.0	12.5 ±1.0	1.11 ± 0.03	good
F7	1:0.5	31 ± 0.6	11.5 ±1.0	1.11 ± 0.09	good
F8	1:0.25	31 ± 0.5	11.2 ± 0.6	1.10 ± 0.05	good

**Table 2:** Results of the prepared formulations’ weight uniformity, hardness, friability, and disintegration time.

Formula Code	Ratio of FEB:CHR	Weight Uniformity (mg ± SD) N = 20	Hardness (Kg/cm^2^) N = 3	Friability (%)	Disintegration Time (min) N = 6
F1	1:2	397 ± 2	3.5 ± 1.2	1.3	2.5 ± 0.5
F2	1:1.75	398 ± 1.5	3.6 ± 1.3	1.2	2.6 ± 0.6
F3	1:1.5	401 ± 2	3.9 ± 0.95	1.25	3.0 ± 1.0
F4	1:1.25	400 ± 2.3	4.0 ± 1.5	1.0	3.2 ± 1.0
F5	1:1	398 ± 2.1	5.2 ± 1.8	1.0	4.1 ± 1.2
F6	1:0.75	402 ±1.6	6.1 ± 1.9	0.78	4.0 ± 0.8
F7	1:0.5	403 ± 2.3	7.0 ± 1.0	0.65	4.0 ± 0.9
F8	1:0.25	401 ± 1.9	7.5 ± 1.2	0.65	4.5 ± 0.75

**Table 3:** Correlation coefficient (R^2^) values of fitting of the FEB release from all formulations.

Mode/Formula Code	Zero-Order (Cum % Drug Release vs. Time)	First-Order (Log (Cum % Drug Release vs. Time)	Peppas Model (Log (Cum % Drug Release vs. Time)	Higuchi Model (Cum % Drug Release vs. SQRT of Time)	Hixson–Crowell Model W0 ^(1/3)^–Wt ^(1/3)^ vs. Time
F1	0.835	0.841	0.972	0.941	0.835
F2	0.794	0.797	0.962	0.914	0.793
F3	0.746	0.749	0.935	0.873	0.746
F4	0.969	0.975	0.990	0.983	0.969
F5	0.972	0.980	0.996	0.989	1.00
F6	0.972	0.990	0.979	0.989	0.972
F7	0.994	0.995	0.987	0.970	0.994
F8	0.986	0.977	0.986	0.968	0.999

**Table 4:** Pharmacokinetic parameters (PKPs) of FEB in rats’ plasma from F8 and the reference formula.

The Formula	Cmax (ng/mL)	AUC 0–12 (ng·mL/h)	AUC Total (ng·mL/h)	Tmax (h)
Reference Formula	150 ± 25	665.5 ± 120	673.04 ± 180	2.00 ± 1.0
F8	155 ± 38	* 1134.5 ± 200	* 1294.24 ± 320	3.00 ± 0.5

* significant on 95% CI.

**Table 5:** The suggested formulations of FEB, showing the FEB:CHR ratios and other excipients.

Formula Code	Ratio of FEB:CHR	FEB (mg)	CHR (mg)	Avecil 102 (mg)	Sodium Starch Glycolate (mg)	Magnesium Stearate (mg)	Total Weight of the Tablet
F1	1:2	80	160	146	10	4	400
F2	1:1.75	80	140	166	10	4	400
F3	1:1.5	80	120	186	10	4	400
F4	1:1.25	80	100	206	10	4	400
F5	1:1	80	80	174	10	4	400
F6	1:0.75	80	60	246	10	4	400
F7	1:0.5	80	40	266	10	4	400
F8	1:0.25	80	20	286	10	4	400

## Data Availability

All of the data are included in the article.
